# Supplementary material for: Factors Associated with Behavioral and Psychological Symptoms of Dementia during COVID-19
Source: Int J Environ Res Public Health. 2022 Aug 15;19(16):10094. doi: 10.3390/ijerph191610094 (PMC9407936; doi:10.3390/ijerph191610094)
Supplement: Supplementary file 1 [file ijerph-19-10094-s001.zip › ijerph-1801961-supplementary.pdf]

Supplement Table S1. Proportion of patients with behavioral disorders in each item of DBD: before and during COVID-19 pandemic, by MCI and Dementia group

	Total Group			MCI Patients			Dementia Patients		
	Before	During	*P* value	Before	During	*P* value	Before	During	*P* value
01. Asks the same question over and over again	1475 (82.9)	484 (84.0)	0.559	531 (71.9)	185 (78.1)	0.073	944 (90.7)	299 (88.2)	0.222
02. Loses, misplaces, or hides things	1263 (71.0)	416 (72.3)	0.581	423 (57.2)	143 (60.3)	0.444	840 (80.8)	273 (80.8)	1.000
03. Shows lack of interest in daily activities	874 (49.4)	288 (50.4)	0.712	245 (33.2)	78 (33.2)	1.000	629 (61.0)	210 (62.5)	0.672
04. Wakes up at night for no obvious reason	266 (15.2)	113 (19.9)	**0.010**	43 (5.9)	24 (10.2)	**0.033**	223 (21.0)	89 (26.7)	0.082
05. Makes unwarranted accusations	321 (18.1)	118 (20.5)	0.214	84 (11.4)	34 (14.3)	0.267	237 (22.9)	84 (24.9)	0.502
06. Sleeps excessively during the day	687 (38.8)	245 (42.6)	0.117	197 (26.7)	79 (33.3)	0.060	490 (47.4)	166 (49.1)	0.636
07. Paces up and down	171 (9.7)	70 (12.2)	0.096	28 (3.8)	6 (2.5)	0.473	143 (13.8)	64 (19.0)	**0.027**
08. Repeats the same action over and over again	239 (13.5)	81 (14.1)	0.751	50 (6.8)	16 (6.8)	1.000	189 (18.3)	65 (19.3)	0.739
09. Is verbally abusive, curses	287 (16.2)	104 (18.1)	0.308	81 (11.0)	39 (16.5)	**0.034**	206 (19.8)	65 (19.2)	0.872
10. Dresses inappropriately	305 (17.2)	102 (17.8)	0.778	56 (7.6)	21 (8.9)	0.606	249 (24.0)	81 (24.0)	1.000
11. Cries or laughs inappropriately	108 (6.1)	46 (8.0)	0.123	11 (1.5)	9 (3.8)	0.055	97 (9.3)	37 (11.0)	0.430
12. Refuses to be helped with personal care	354 (19.9)	124 (21.6)	0.435	100 (13.6)	37 (15.6)	0.492	254 (24.5)	87 (25.7)	0.698
13. Hoards things for no obvious reason	410 (23.1)	151 (26.4)	0.127	103 (14.0)	47 (19.9)	0.035	307 (29.6)	104 (30.9)	0.720
14. Moves arms or legs in a restless or agitated way	126 (7.1)	35 (6.1)	0.471	36 (4.9)	9 (3.8)	0.626	90 (8.7)	26 (7.7)	0.650
15. Empties drawers or closets	134 (7.5)	48 (8.4)	0.567	14 (1.9)	6 (2.6)	0.720	120 (11.6)	42 (12.5)	0.731
16. Wanders in the house at night	88 (5.0)	38 (6.7)	0.154	9 (1.2)	2 (0.9)	0.917	79 (7.7)	36 (10.7)	0.110
17. Gets lost outside	89 (5.0)	43 (7.5)	**0.031**	6 (0.8)	5 (2.1)	0.190	83 (8.0)	38 (11.3)	0.085
18. Refuses to eat	72 (4.1)	23 (4.0)	1.000	12 (1.6)	4 (1.7)	1.000	60 (5.8)	19 (5.7)	1.000
19. Overeats	267 (15.0)	82 (14.30	0.730	79 (10.7)	21 (8.9)	0.509	188 (18.2)	61 (18.2)	1.000
20. Is incontinent of urine	229 (12.9)	77 (13.4)	0.809	40 (5.4)	17 (7.2)	0.389	189 (18.3)	60 (17.8)	0.897
21. Wanders aimlessly outside or in the house during the day	79 (4.5)	39 (6.8)	**0.033**	4 (0.5)	1 (0.4)	1.000	75 (7.2)	38 (11.3)	**0.026**
22. Physical attacks	44 (2.5)	22 (3.8)	0.117	8 (1.1)	7 (3.0)	0.081	36 (3.5)	15 (4.4)	0.513

23. Screams for no reason	47 (2.6)	17 (3.0)	0.790	12 (1.6)	5 (2.1)	0.826	35 (3.4)	12 (3.6)	1.000
24. Makes inappropriate sexual advances	13 (0.7)	2 (0.3)	0.484	8 (1.1)	0 (0.0)	0.234	5 (0.5)	2 (0.6)	1.000
25. Exposes himself/herself indecently	5 (0.3)	1 (0.2)	1.000	0 (0.0)	0 (0.0)	1.000	5 (0.5)	1 (0.3)	1.000
26. Destroys property or clothing	21 (1.2)	5 (0.9)	0.699	3 (0.4)	1 (0.4)	1.000	18 (1.7)	4 (1.2)	0.653
27. Is incontinent of feces	85 (4.8)	32 (5.6)	0.519	10 (1.4)	6 (2.5)	0.338	75 (7.2)	26 (7.7)	0.872
28. Throws food	15 (0.8)	2 (0.3)	0.348	4 (0.5)	1 (0.4)	1.000	11 (1.1)	1 (0.3)	0.328

* For each item of the DBD, “positive for the behavioral disorder” was defined as an answer of "sometimes”, “often”, or “always," and the proportion was calculated. *P* values in **bold** indicate statistical significance. Abbreviations: DBD = Dementia Behavioral Disturbance Scale.
